# Pathophysiological mechanisms explaining poor clinical outcome of older cancer patients with low skeletal muscle mass

**DOI:** 10.1111/apha.13516

**Published:** 2020-07-24

**Authors:** Stéphanie M. L. M. Looijaard, Miriam L. te Lintel Hekkert, Rob C. I. Wüst, René H. J. Otten, Carel G. M. Meskers, Andrea B. Maier

**Affiliations:** ^1^ Department of Human Movement Sciences @AgeAmsterdam Faculty of Behavioural and Movement Sciences Vrije Universiteit Amsterdam Amsterdam Movement Sciences Amsterdam The Netherlands; ^2^ University Library Vrije Universiteit Amsterdam Amsterdam The Netherlands; ^3^ Department of Rehabilitation Medicine Amsterdam University Medical Center VU University Medical Center Amsterdam Movement Sciences Amsterdam The Netherlands; ^4^ Department of Medicine and Aged Care @AgeMelbourne The Royal Melbourne Hospital University of Melbourne Melbourne Australia

**Keywords:** aged, cachexia, geriatric oncology, neoplasms, physiopathology, sarcopenia

## Abstract

Low skeletal muscle mass is highly prevalent in older cancer patients and affects 5% to 89% depending on the type and stage of cancer. Low skeletal muscle mass is associated with poor clinical outcomes such as post‐operative complications, chemotherapy toxicity and mortality in older cancer patients. Little is known about the mediating pathophysiological mechanisms. In this review, we summarize proposed pathophysiological mechanisms underlying the association between low skeletal muscle mass and poor clinical outcomes in older cancer patients including a) systemic inflammation; b) insulin‐dependent glucose handling; c) mitochondrial function; d) protein status and; e) pharmacokinetics of anticancer drugs. The mechanisms of altered myokine balance negatively affecting the innate and adaptive immune system, and altered pharmacokinetics of anticancer drugs leading to a relative overdosage of anticancer drugs are best‐substantiated. The effects of glucose intolerance and circulating mitochondrial DNA as a consequence of low skeletal muscle mass are topics of interest for future research. Restoring myokine balance through physical exercise, exercise mimetics, neuro‐muscular activation and adapting anticancer drug dosing on skeletal muscle mass could be targeted approaches to improve clinical outcomes in older cancer patients with low skeletal muscle mass.

## 1 INTRODUCTION

Ageing is associated with loss of skeletal muscle mass^1^ and strength.^2^ Sarcopenia is diagnosed if muscle mass and strength fall below a certain threshold.^3^ Approximately 10% of the older population suffer from sarcopenia,^4^ but the prevalence is higher in patients with cancer and other age‐related diseases.^5^, ^6^ The prevalence rate of sarcopenia in patients with cancer was estimated at 38.6%,^7^ varying between 5% and 89% depending on the type and stage of cancer^8^ and on the applied diagnostic criteria for sarcopenia.^9^, ^10^, ^11^ In cancer patients, sarcopenia can co‐occur with cachexia, which is characterized by severe weight loss and loss of skeletal muscle and adipose tissue.^12^ The prevalence of cachexia highly depends on the underlying disease, but between 50% and 80% of patients with advanced malignant cancers are thought to suffer from cachexia.^13^ Although considered two separate diseases, the pathophysiology of sarcopenia and cachexia are overlapping, both are multifactorial and include a misbalance between lower protein synthesis and higher protein degradation because of an elevated intracellular inflammation and oxidative stress.^14^, ^15^, ^16^

Low skeletal muscle mass is often perceived as a biomarker for deprived fitness and health status, which can lower the resilience to stressors that accompany cancer and cancer treatment.^17^, ^18^, ^19^, ^20^, ^21^, ^22^ Low skeletal muscle mass in cancer patients has been associated with poor clinical outcomes including higher post‐operative complication rates,^7^, ^23^ higher chemotherapy toxicity,^7^, ^8^, ^23^ lower disease‐free or progression‐free survival^7^, ^8^, ^23^ and higher overall mortality,^7^, ^8^, ^23^, ^24^ although associations are not considered to be straightforward.^25^ Systemic inflammation, insulin‐dependent glucose handling and alterations in energy‐ and protein metabolism and pharmacokinetics have been proposed as pathophysiological mechanisms explaining the association between low skeletal muscle mass and poor clinical outcomes in older patients with cancer.^19^, ^24^, ^26^ If and how these mechanisms contribute to clinical outcomes is currently unknown. Understanding the pathophysiological consequences of low skeletal muscle mass on clinical outcomes and disease progression offers new directions for interventions in older cancer patients.

**Figure 1:**
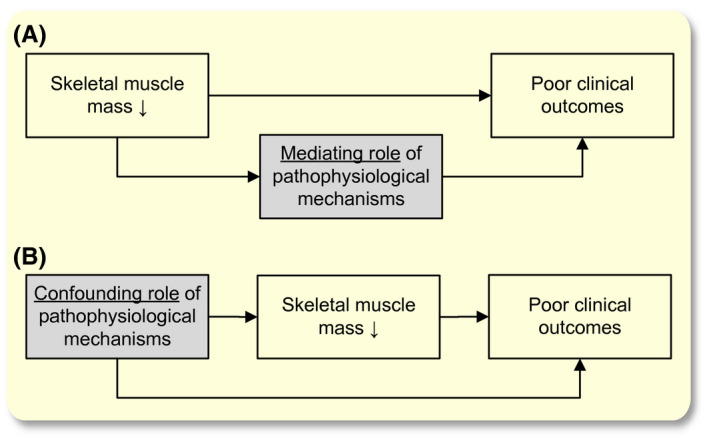
Pathophysiological mechanisms underlying the association between low skeletal muscle mass and poor clinical outcomes by mediation 1(a) and confounding 1(b) in older cancer patients using directed acyclic graphs

**Figure 2:**
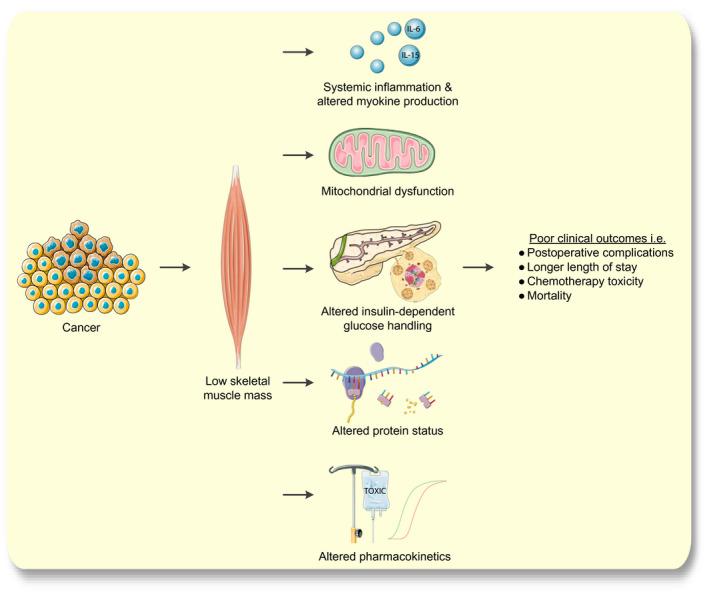
Theoretical framework and overview of the scope of the review

This review provides an overview and discussion of the described pathophysiological mechanisms in the literature that could underlie the association between low skeletal muscle mass and poor clinical outcomes in older cancer patients. We specifically focus on the pathophysiological consequences of low skeletal muscle mass as illustrated in the directed acyclic graph in Figure 1.^27^ We will describe: a) the role of skeletal muscle mass to modulate the immune system through cytokines and myokines including the effects of physical activity; b) the influence of low skeletal muscle mass on insulin‐dependent glucose handling and c) mitochondrial function; d) the effects on whole‐body protein status; e) pharmacokinetics of anticancer drugs. Figure 2 provides an illustrated overview of the scope of the article. The literature search is presented in the Appendix. We conclude by exploring future directions for research and potential interventions that could decrease the risk of poor clinical outcomes in older cancer patients with low skeletal muscle mass.

## 2 OVERVIEW OF PATHOPHYSIOLOGICAL MECHANISMS

**Figure 3:**
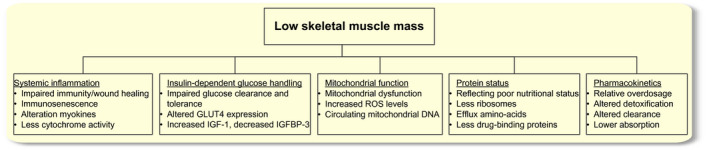
Pathophysiological mechanisms and markers potentially underlying the association between low skeletal muscle mass and poor clinical outcomes in older cancer patients

Figure 3 provides a summarized overview of the mechanisms that potentially play a role in the association between low skeletal muscle mass and poor clinical outcomes in older cancer patients. These mechanisms will be discussed extensively throughout this narrative review.

## 3 SYSTEMIC INFLAMMATION

Skeletal muscle fibres are able to actively shape the immune system in both a pro‐ and anti‐inflammatory manner, regulating innate and adaptive immune responses.^28^, ^29^ In this way, low skeletal muscle mass directly contributes to chronic low‐grade local and systemic inflammation.^22^, ^30^, ^31^ Various clinical observational studies showed significant associations between low skeletal muscle mass and higher inflammatory markers, such as a higher neutrophil‐to‐lymphocyte ratio and higher C‐reactive protein levels, in older cancer patients.^32^, ^33^, ^34^, ^35^, ^36^, ^37^ This dose‐response relation between low skeletal muscle mass and systemic inflammation was independent of cancer stage, age and sex.^37^ These inflammatory markers are significantly associated with overall^34^, ^38^, ^39^, ^40^ and cancer‐specific^38^, ^39^ mortality. Patients with a combination of low skeletal muscle mass and high inflammatory markers had higher mortality rates than patients with low skeletal muscle mass and low inflammatory markers.^37^, ^41^ Therefore, it remains elusive whether systemic inflammation is an additional or mediating mechanism for poor clinical outcomes in cancer patients. Figure 3 includes a summary of pathophysiological mechanisms caused by systemic inflammation that may explain the association between low skeletal muscle mass and poor clinical outcomes.

Patients with low skeletal muscle mass at the time of hospital admission have a doubled risk of nosocomial infections during the first weeks of hospitalization^42^ and a higher risk of post‐operative complications, requiring inpatient rehabilitation and longer hospital stay.^43^ This could potentially be caused by impaired wound healing^44^ or by the effect of low skeletal muscle mass on specific muscle function such as breathing or swallowing. For example atrophy and weakness in the diaphragm muscle could lead to respiratory dysfunction. The resulting decreased airflow and inability to fully inflate the lung and cough facilitates the development of pneumonia.^45^, ^46^ Indeed, muscle wasting in colon‐26‐bearing mice caused significant atrophy in the diaphragm muscle, which resulted in a lower tidal volume and an inability to increase breathing frequency and tidal volume during a respiratory challenge.^45^ Also, a low mass and function of muscles involved in swallowing can lead to dysphagia, which increases the risk of complications such as aspiration pneumonia.^47^, ^48^ Furthermore, systemic inflammation is known to induce chemotherapy toxicity (see paragraph ‘pharmacokinetics of anticancer drugs’) and aggravate the already impaired immune function.^44^, ^49^ Impaired immune function combined with increased inflammatory cytokines contributes to immunosenescence,^29^ which could increase the risk of additional complications.^50^ Increased tumour aggressiveness,^37^ poor treatment response^37^ and a higher risk of cancer development^21^ have also been attributed to an increased inflammatory status.

### 3.1 Myokine secretion and physical exercise

Based on the suggestion that skeletal muscle acts as an endocrine organ,^51^ one of the predominant, most described and best‐substantiated theories is that low skeletal muscle mass results in less myokine production. Myokines are small molecules released by contracting skeletal muscle, which can exert autocrine, paracrine and endocrine effects on other tissues.^51^ More than 200 myokines have been discovered so far, but their individual functions are still mostly unknown. Overall, alterations in the balance between myokines and adipokines can negatively influence the innate and adaptive immune system.^52^ In the context of exercise and cancer immunology, interleukin (IL)‐15 and IL‐6 have been studied extensively and modulate the innate and adaptive immune system.^51^ IL‐15 is involved in the regulation of natural killer cell number and activity and protects natural killer cells from apoptosis.^29^, ^52^ IL‐15 knockout mice had almost no mature natural killer cells and natural killer cells were destroyed after being transferred into the same knockout mice.^52^, ^53^, ^54^ Lower IL‐15 release into the bloodstream as a consequence of low skeletal muscle mass has thus been proposed to lead to lower natural killer cell number and survival,^52^ increasing the risk of infectious complications^55^ and shortening survival^56^ in cancer patients. Moreover, IL‐15 is involved in CD8 T‐cells homeostasis, the survival of naive T‐cells and proliferation of B‐cells.^29^, ^57^ The first clinical trial in patients with metastatic melanoma or renal cell cancer showed that infusions of IL‐15 led to redistribution and hyperproliferation of natural killer cells and CD8 memory T‐cells.^57^ Although grade 3 toxicities were observed, lower dosages of IL‐15 could safely be administered. This allows targeted interventions on myokine infusions to be tested as potential new strategies in anticancer treatment.^57^

IL‐6 represents another myokine which is expressed in high levels in skeletal muscle tissue. IL‐6 exerts pro‐inflammatory effects in response to pathogens, including T‐cell recruitment and promoting antibody production from B‐cells.^29^ As a myokine, IL‐6 has been indicated to play an important role in the redistribution and infiltration of natural killer cells, thereby suppressing tumour growth.^58^ As low skeletal muscle mass is related to lower levels of IL‐6, low muscle mass could inhibit the suppression of tumour progression, worsening the prognosis of cancer patients.^58^, ^59^, ^60^

Research on the effects of prescribing exercise in oncological patients is rapidly expanding,^61^, ^62^, ^63^ particularly after it was observed that voluntary running in tumour‐bearing mice suppressed tumour growth, likely by enabling IL‐6‐sensitive natural killer cells to infiltrate tumour tissue.^58^, ^64^ Physical exercise itself was proved to be the crucial factor to evoke the effects of IL‐6 on tumour growth, as simply administering an IL‐6 injection did not have similar repressing effects.^58^ This suggests that likely a combination of currently known and unknown other myokines can explain these adaptations. As such, the main current hypothesis on how exercise prevents and suppresses the development of cancer is that exercise alters the host immune system, via exercise‐induced factors (including myokines and other mobilizing serum factors) released in the bloodstream.^65^ These positive effects of exercise on immune function have been corroborated in cancer patients, demonstrated by an increase in natural killer cell cytotoxic activity, lymphocyte proliferation and number of granulocytes after chronic aerobic and/or resistance exercise.^66^

Other myokines worth mentioning are IL‐8 and myostatin. IL‐8 expression is elevated in cancer cachexia^67^ and higher IL‐8 expression has a signalling role in the tumour microenvironment, it induces angiogenesis and stimulates tumour growth.^67^, ^68^ Myostatin, also known as growth differentiation factor 8, negatively correlates with muscle mass. In case of a muscle wasting disease, its expression is increased.^67^, ^69^ Myostatin might be secreted from primary tumours, but its precise role in tumour metabolism remains unknown.^69^ Myokines such as myonectin, decorin and fibroblast growth factor 21 possibly also link muscle mass to cancer outcome, but are less studied in this context.^67^

Since exercise is accompanied by an increase in the blood concentration of a large number of myokines,^70^ other myokines likely contribute as well. Indeed, oncostatin M^65^, ^71^ and irisin^65^, ^72^ showed direct anti‐proliferative effects on cancer cells in breast cancer cells. Osteonectin, also known as secreted protein acidic and rich in cysteine (SPARC), has similar effects in colon cancer cells.^65^, ^73^ Although the function of many myokines remains unknown, some of them have already shown to have therapeutic potential to (in)directly improve clinical outcomes in cancer patients.^64^, ^70^ Exercise‐induced alteration in immune function, likely through the secretion of myokines and other mobilizing serum factors, are potentially novel targets and represent promising new directions for treatment options for patients with cancer. Also, exercise mimetics such as musclin are currently receiving a lot of attention in the exercise physiology field. These ‘exercise pills’ could potentially be of great use for cancer patients who are unable to perform (strenuous) exercise.^74^ Non‐pharmacological interventions such as neuro‐muscular electrical stimulation could also be given to patients who are unable to exercise. Two controlled studies in patients with advanced solid cancers showed that a twelve week program with two sessions of neuro‐muscular electrical stimulation per week combined with individualized nutritional support, led to a significantly higher muscle mass and physical performance at the end of the intervention compared to the control group that only received individualized nutritional support.^75^, ^76^ These fields of research will likely receive major attention in the coming years.

It should be emphasised that other indirect exercise‐induced adaptations may contribute as well to the observed effects. In this review, we highlight the role of skeletal muscle in the pathophysiology of clinical outcome measures, and it is likely that exercise‐induced maintenance or increase in muscle mass and oxidative capacity per se contribute to understanding the underlying mechanisms. Indeed, a 16‐week high‐intensity exercise training intervention in breast cancer patients was found to maintain or increase muscle citrate synthase activity, size and capillarization of both slow‐switch (type I) and fast‐twitch glycolytic (type II) fibres.^77^ Similar as during the process of ageing, mainly fast‐twitch glycolytic fibres (type II) are lost during cancer cachexia and may even lead to a fast‐to‐slow fibre type shift.^78^ These alterations were associated with self‐reported fatigue, confirming the notion that factors independent of the immune system contribute to improved clinical outcome in cancer patients.^77^ Engaging in physical exercise during systemic anticancer treatment can possibly limit the disruption that anticancer drugs cause on molecular signalling pathways.^79^ Recent advances have suggested a role for HIF1α in the development of cancer cachexia and exercise‐induced alterations in skeletal muscle function, but more work is needed to fully understand these mechanisms.^80^ Clearly, more experimental work is necessary to fully understand the contributing role for exercise in preventing and suppressing development of cancer growth in various types of cancer.

## 4 INSULIN‐DEPENDENT GLUCOSE HANDLING AND TUMOUR GROWTH

Skeletal muscle has a primary role in insulin‐mediated glucose metabolism as it is the main target organ of insulin‐dependent glucose uptake.^81^ In the case of atrophying skeletal muscle, lipids accumulating in muscle tissue can induce glucose intolerance through insulin resistance.^82^ On the other hand, glucose intolerance and insulin resistance have long been recognized as a manifestation of cancer.^83^, ^84^ Insulin resistance was found to be associated with overall and cancer‐specific survival^85^, ^86^ and post‐operative complications.^36^ Pathophysiological mechanisms caused by alterations in insulin‐dependent glucose handling that may relate to poor clinical outcomes in cancer patients are summarized in Figure 3. Interestingly, the expression of the insulin‐regulated glucose transporter, GLUT4, is reported to increase during anticancer drug treatment,^79^ but is it unknown what the underlying mechanisms are of these alterations. Since tumour tissue is also known to take up glucose, a lower glucose clearance must be sought in alterations in insulin sensitivity in other organs.^87^

This whole‐body insulin resistance might simply be because of a lower skeletal muscle mass in cachectic cancer patients,^88^, ^89^, ^90^ but other factors likely contribute as well. For instance another mechanism by which low skeletal muscle mass causes and exacerbates insulin resistance is by altering the secretion of insulin sensitivity‐regulating myokines.^88^ Insulin resistance could lead to increased levels of insulin‐like growth factor 1 (IGF‐1) and decreased levels of insulin‐like growth factor‐binding protein 3 (IGFBP‐3).^91^ Higher IGF‐1 and lower IGFBP‐3 levels are associated with disease progression in patients with prostate cancer.^91^, ^92^, ^93^ Furthermore, a cachexia‐related impaired glucose clearance from the blood allows more glucose to become available for uptake in tumour cells.^94^ Since tumours often rely on glycolysis for cell survival and proliferation,^94^ higher blood glucose levels could accelerate cancer growth and disease progression. Reducing blood glucose levels by caloric restriction or ketogenic diets have recently attracted attention in the literature,^95^ with mixed results and opinions.^96^ Clearly, such dietary interventions can accelerate the loss of skeletal muscle mass which would not be without consequences. Further research will be needed to clarify the role of low skeletal muscle mass‐induced alterations in insulin resistance and insulin‐like growth factors in the progression of cancer.

## 5 MITOCHONDRIAL FUNCTION

A high skeletal mitochondrial function is generally associated with a higher endurance capacity and a lower sense of fatigue during submaximal exercise. As a result, an impaired skeletal mitochondrial function can directly explain an increased feeling of fatigue in patients with cancer.^97^ Cancer progression, as well as anticancer drugs are both known to negatively affect skeletal muscle mitochondrial function.^97^ Mitochondrial abnormalities are common in sarcopenia^98^, ^99^ and cancer cachexia.^100^, ^101^, ^102^, ^103^ In Figure 3 the potential pathophysiological mechanisms caused by mitochondrial dysfunction that may affect poor clinical outcomes in cancer patients are highlighted. In particular, disturbed mitochondrial dynamics, mitophagy and an impaired mitochondrial biogenesis are observed in cancer cachexia, all reducing oxidative phosphorylation capacity and increasing reactive oxygen species (ROS) production.^101^ These processes likely contribute to the development of muscle wasting in patients with cancer.^100^ At the same time, various anticancer drugs are known to non‐specifically induce skeletal muscle mitochondrial dysfunction.^104^ For instance doxorubicin is known to accumulate inside mitochondria and induces mitochondrial complex I dysfunction, reducing adenosine‐5’‐triphosphate (ATP) synthesis rates and producing ROS,^102^, ^104^ ultimately reducing muscle size and function by DNA damage, protein oxidation and apoptosis.^101^ Other chemotherapeutics have similar effects,^97^ and can modulate mitochondrial DNA (mtDNA). Clearly, the combination of cancer and current anticancer therapies induces mitochondrial damage and ultimately leads to a vicious circle further deteriorating skeletal muscle mass and function.^101^

More recent evidence hints to an additional role of mitochondria in the pathophysiology of skeletal muscle wasting‐induced cancer progression. When mitochondria are defective and are broken down during mitophagy, fragments of mtDNA can be found in the circulation. A high level of circulating mtDNA is linked to a faster cancer progression and poor survival of patients with ovarian cancer.^105^, ^106^ It remains unclear whether these mtDNA fragments come from the tumour itself or from non‐tumour tissue, although recent evidence hints towards the latter.^107^ As skeletal muscle tissue is rich in mitochondria, skeletal muscle wasting might be a source of circulating mtDNA.^108^ The underlying molecular mechanism is currently unknown, but two options are plausible. The first one is that a high level of circulating mtDNA serves as a biomarker for high muscle breakdown rates and severe cachexia. Hence, the poor survival rates linked to high circulating mtDNA can be explained by complications because of high muscle breakdown rates. An alternative mechanism is that circulating mtDNA (and other mitochondria‐derived molecules) can act as damage‐associated molecular pattern (DAMP) molecules and therefore affect distant organ function, including immune function.^109^ Circulating mtDNA can activate neutrophil and platelet responses facilitating tumour metastasis and obstructing anti‐tumour immunity.^110^ This field is vastly unknown and future research will be required to elucidate the underlying mechanisms, clinical contribution and therapeutic potential.

## 6 LOW PROTEIN STATUS AND POOR NUTRITIONAL STATUS

An important contributing mechanism to the development of low skeletal muscle mass is protein status alteration. Muscle protein synthesis rate is determined by the overall health status, nutrient availability and physical activity.^111^ Low nutrient intake and low levels of muscle activation lead to decreased protein anabolism and increased protein catabolism, which negatively affect skeletal muscle mass in animal models^112^ and in human research studies.^111^ In case of low skeletal muscle mass and low muscle activation, protein synthesis and function are repressed.^113^ The effects of muscle activation are further described in the paragraph on ‘myokine secretion and physical activity’. Clinical studies quantifying protein status by albumin levels, have established hypoalbuminaemia to be associated with measures of sarcopenia,^114^ post‐operative complications and longer length of hospital stay.^115^, ^116^ As low skeletal muscle mass is also predictive of post‐operative complications and overall survival independent of albumin status,^50^, ^55^, ^117^ the mediating role of overall protein status in the association between low skeletal muscle mass and poor clinical outcomes in cancer patients is not conclusive. Potential explaining pathophysiological mechanisms are summarized in Figure 3.

It is widely accepted that low protein status is a reflection of a poor nutritional status, which is prognostic for poor clinical outcomes in cancer patients.^50^, ^117^, ^118^ Questions have been posed whether serum albumin levels are a proper marker of nutritional status because of the low diagnostic accuracy.^117^, ^119^ On the other hand, protein synthesis occurs in the liver where ribosomes are most predominantly present, but also takes place in skeletal muscle fibres.^120^ Hence, low skeletal muscle mass is accompanied by fewer ribosomes, leading to lower absolute protein synthesis rates,^121^ which might have negative systemic effects and influence clinical outcomes. Another theory is that breakdown of muscle proteins leads to efflux of stored amino acids into the bloodstream,^36^ which then becomes available for take‐up by the tumour to promote tumour growth.^122^ Moreover, low protein status affects the risk of chemotherapy toxicity (see paragraph ‘pharmacokinetics of anticancer drugs’).

## 7 PHARMACOKINETICS OF ANTICANCER DRUGS

Pharmacokinetics play an important role in patients with cancer since the majority of patients are treated with systemic therapies such as chemotherapy. Pathophysiological mechanisms caused by an alteration in pharmacokinetics of anticancer drugs because of low skeletal muscle mass that may increase the risk of poor clinical outcomes in cancer patients are highlighted in Figure 3. Over the past decades, dosing of anticancer drugs such as chemotherapy has been based on total body surface area, a constitute of body weight and height.^123^ As basing dosage on body surface area did not reduce interpatient variability in drug clearance^124^ or the prevalence of dose‐limiting toxicity,^125^ it has been questioned whether body surface area is the appropriate measure to determine drug dosage. Dosing chemotherapy protocols based on body surface area led to a higher dosage of chemotherapy per kilogram lean body or skeletal muscle mass, which in turn was associated with chemotherapy toxicity.^126^, ^127^ The so‐called ‘overdosage hypothesis’ states that basing treatment dosage on body surface area leads to a relative overdosing of treatment in patients with low skeletal muscle mass because of a lower area and volume of distribution of drugs,^26^ and has been recalled by many others in the oncological field.^19^, ^22^, ^36^, ^126^, ^127^ Therefore, lean body mass has been suggested to be used to individualize treatment dosage. This is of even more importance in hydrophilic agents that are mainly metabolized and distributed in lean tissue.^26^ In addition, detoxification pathways of specific chemotherapeutics partly occur in skeletal muscles. For example anthracyclines such as doxorubicin are metabolized in the electron transport chains of mitochondria which are present in high concentrations in skeletal muscle tissue.^104^ The level of sequestering of doxorubicin in skeletal muscle influences its systemic availability and rate and amount of detoxification.^128^

Next to the decreased distribution of chemotherapeutics, clearance might be altered in cancer patients with low skeletal muscle mass. Patients with low skeletal muscle mass were found to have a higher area under the curve (AUC) and lower plasma clearance of multiple chemotherapeutics compared to patients with normal skeletal muscle mass.^36^, ^126^, ^127^ Patients with low skeletal muscle mass and low clearance also had a higher risk of chemotherapy toxicity.^129^, ^130^ On the other hand, the association between skeletal muscle mass and plasma clearance^131^, ^132^, ^133^ and the association between plasma clearance and chemotherapy toxicity^20^, ^132^, ^133^ could not always be confirmed. As the current body of literature shows inconsistencies, further research investigating the link between altered clearance and the association between low skeletal muscle mass and poor clinical outcomes in cancer patients is necessary.

Another process of pharmacokinetics is the absorption of anticancer drugs. Low skeletal muscle mass in cancer patients is accompanied by an increase in permeability of the gut barrier, causing a leakage of endotoxins into the systemic circulation evoking a low‐grade systemic inflammatory response.^134^, ^135^ Moreover, anticancer drugs could cause the tight junctions in the intestinal tissues to become weaker and therewith further induce gut barrier dysfunction.^134^ The resulting increase in leakage of anticancer drugs into intestinal tissues and the systemic circulation might increase the risk of toxicity of anticancer drugs.^36^, ^134^

Other roles of how low skeletal muscle mass affects pharmacokinetics are via inflammation and overall protein status. The low‐grade inflammatory state that accompanies low skeletal muscle mass leads to a decrease in liver cytochrome activity.^136^, ^137^, ^138^ The resulting lower metabolic capacity of the liver increases the exposure to chemotherapeutics and causes toxicity.^19^, ^20^, ^36^, ^130^ Because of a lower skeletal muscle mass, less skeletal muscle proteins might be available for potential protein‐binding of chemicals, also increasing exposure to chemotherapeutics and the risk of toxicity.^20^, ^36^, ^130^, ^131^, ^139^ In addition, the concentration and activity of dihydropyrimidine dehydrogenase (DPD) are thought to decrease as a consequence of low protein status. Particular chemotherapeutics that are metabolized by DPD, such as 5‐fluorouracil, could consequently accumulate in the bloodstream, leading to increased toxicity.^36^ Countering an influence of low protein status on pharmacokinetics and risk of toxicity, low protein levels were not associated with more unbound chemotherapeutic in patients with hepatic dysfunction.^140^ However, apart from low protein serum levels, low skeletal muscle mass itself could contribute to less drug‐binding and higher exposure to anticancer drugs as protein‐binding also occurs in skeletal muscle tissue.^131^

Skeletal muscle mass was predominantly measured using bio‐impedance analysis derived lean body mass in the aforementioned pharmacokinetics studies. Bio‐impedance analysis is considered a valid tool to for the assessment of total body and segmental body composition.^141^ As lean body mass not only includes skeletal muscle mass but also organs, bones and inter‐ and intracellular water, other tissues such as the liver could have also contributed to the absorption, distribution and metabolism of anticancer drugs. However, as clearance of chemotherapeutics cannot be fully explained by liver volume or liver metabolism, skeletal muscle mass is expected to contribute to drug metabolism.^131^, ^142^, ^143^

## 8 REVERSE CAUSATION: TUMOUR CAUSING SKELETAL MUSCLE DYSFUNCTION

The majority of this review is based on associations only, as longitudinal studies assessing the association between low skeletal muscle mass, pathophysiological mechanisms and poor clinical outcomes in cancer patients are scarce. Thus, mediating roles of pathophysiological mechanisms cannot be substantiated firmly, as they can also reversely affect skeletal muscle mass.^15^, ^16^, ^29^ Mutual influence is most likely,^37^ which limits the ability to determine causality. Another factor is the interplay with cancer and anticancer treatment, as both can influence skeletal muscle mass, systemic inflammation, insulin‐dependent glucose handling, protein status and pharmacokinetics of anticancer drugs.^22^, ^144^ The definition of cachexia incorporates the negative influence of a high‐demanding metabolic disease on skeletal muscle mass.^12^ As more aggressive tumours have a higher metabolic demand, low skeletal muscle mass could also be an indicator of more aggressive cancers^59^, ^145^, ^146^ or of tumour progression,^147^ and thus negatively affects clinical outcomes. Moreover, tumour‐produced cytokines can lead to a state of inflammation and can increase insulin resistance.^144^, ^146^ Protein status is often lower becaus of loss of appetite provoked by anticancer treatment and malnutrition caused by the catabolic state of the body.^148^ Although a negative effect of low skeletal muscle mass on clinical outcomes was observed in early‐stage cancers when cancer cachexia is not expected,^22^, ^149^ these pathophysiological mechanisms cannot be seen separately from the influence of cancer disease activity and the influence of anticancer treatment.^22^ It is not clear whether the association between low muscle mass and the risk of poor clinical outcomes is linear or if a critical threshold of muscle mass associates with poor clinical outcomes. Described muscle mass cut‐offs to distinguish patients with a low and high risk of poor clinical outcomes are highly variable and have not been validated yet in older cancer patients.

## 9 LIMITATIONS AND FUTURE DIRECTIONS

This review focussed on low skeletal muscle mass only. Other aspects of sarcopenia and cachexia or the components of muscle failure,^150^ that is muscle strength and physical performance, could also be risk factors for poor clinical outcomes in older cancer patients.^151^ Although sarcopenia and cachexia are separate diseases, the distinction is very difficult in the presence of cancer as they share a common clinical presentation, that is low muscle mass. In non‐longitudinal studies, it is impossible to distinguish whether low skeletal muscle mass is a consequence of age‐related sarcopenia or cancer‐related cachexia. The majority of studies defines these diseases based on low muscle mass, whereas other measures including muscle strength, physical performance, weight loss, fat wasting and metabolic state are required to make the distinction.^3^, ^12^ Reverse causation has to be kept in mind because of the interplay between skeletal muscle mass, pathophysiology and cancer. In addition, it is important that the pathophysiological mechanisms are likely not separate entities but are probably highly interconnected and interact in their influence on poor clinical outcomes in cancer patients.

**Figure 4:**
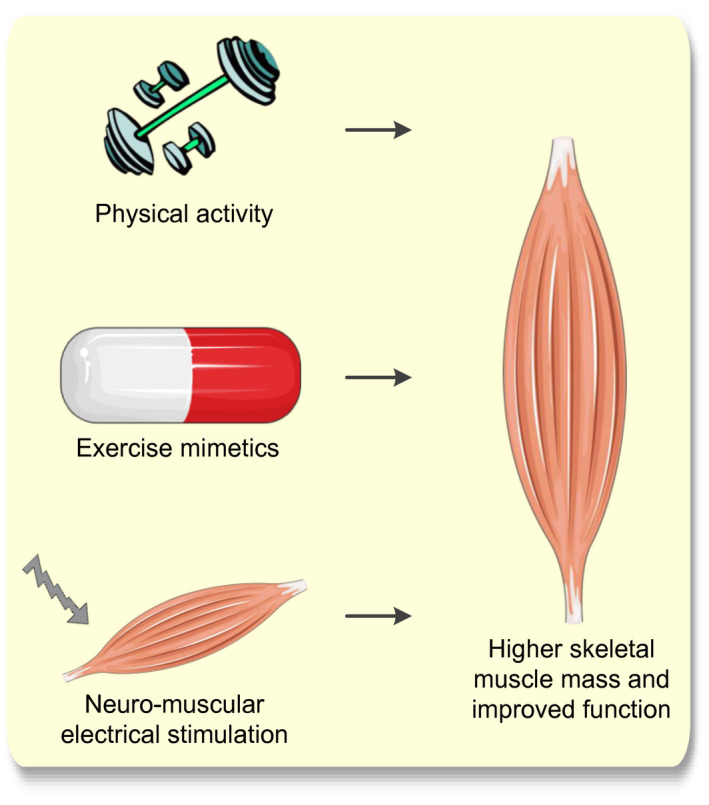
Overview of possible therapeutic interventions to reduce the risk of poor clinical outcomes in older cancer patients

The work presented in this review summarized how low skeletal muscle mass might lead to poor clinical outcomes in older cancer patients. The first step in reducing the risk of poor clinical outcomes in older cancer patients would be to prevent the loss of skeletal muscle mass. Inducing myokine production through physical exercise may act as a therapeutic target to prevent or counteract skeletal muscle mass decline^67^, ^152^ and may prevent its negative effects on clinical outcomes. Voluntary wheel running in mice was able to preserve skeletal muscle mass during anticancer treatment with cisplatin, whereas mice without training lost more than 20% of their lean body mass.^153^ However, while exercise improved skeletal muscle mass in untreated and chemotherapy‐treated tumour‐bearing mice, it worsened survival in late cachexia stages.^154^ Cancer patients with advanced muscle wasting may have passed ‘a point of no return’ in which exercise can become dysfunctional. For cancer patients who are unable to exercise, alternative administration of myokines such as newly developed exercise mimetics or neuro‐muscular electrical stimulation may offer possibilities to reduce the risk of poor clinical outcomes.^74^ If muscle deprivation is already present, targeted interventions to prevent the consequent pathophysiological mechanisms from affecting clinical outcome may be beneficial. The ability of exercise training (aerobic and resistance) and nutritional interventions to reduce inflammation and improve immunity,^66^, ^155^ reduce oxidative stress and insulin resistance,^155^, ^156^ preserve mitochondrial content,^77^ and simultaneously preserve or ameliorate skeletal muscle mass and improve clinical outcomes in cancer patients^157^ has recently been suggested. Figure 4 provides an overview of possible therapeutic interventions to reduce the risk of poor clinical outcomes as a consequence of low muscle mass in older cancer patients. Future research should focus on gaining insight into causality of muscle wasting and poor clinical outcomes by longitudinal, interventional studies during controlled muscle wasting in animal models. Eventually, this should debouch into specific interventions on these mechanisms to improve clinical outcomes in older patients with cancer.

## 10 CONCLUSIONS

In the underpinning of the association of low skeletal muscle mass with poor clinical outcomes in older cancer patients, pathophysiology‐based mechanisms of altered myokine balance affecting the innate and adaptive immune system and altered pharmacokinetics of anticancer drugs leading to a relative overdosage are best‐substantiated. The effects of insulin resistance and circulating mitochondrial DNA as a consequence of low skeletal muscle mass require further exploration. It remains elusive whether these mechanisms are caused by low skeletal muscle mass, and reverse causation should be considered carefully. Developing targeted interventions to restore myokine balance through physical exercise, neuro‐muscular electrical stimulation or exercise mimetics and adapting anticancer drug dosing based on skeletal muscle mass, might be targeted approaches to improve clinical outcomes in older cancer patients with low muscle mass.

## CONFLICTS OF INTEREST

The authors declare that they have no conflict of interest.

## Supporting information

Supplementary Material
