# Supplementary material for: Pathophysiological mechanisms explaining poor clinical outcome of older cancer patients with low skeletal muscle mass
Source: Acta Physiol (Oxf). 2020 Jul 24;231(1):e13516. doi: 10.1111/apha.13516 (PMC7757176; doi:10.1111/apha.13516)
Supplement: Supplementary file 1 — Supplementary Material [file APHA-231-e13516-s001.docx]

**Appendix**

**Literature search**
Bibliographic databases PubMed and EMBASE.com were systematically searched and complemented with references from relevant articles. The search term ‘cancer’ was combined with terms comprising ‘skeletal muscle mass’ and terms for pre-selected pathophysiological mechanisms ‘inflammation’, ‘insulin-dependent glucose handling’, ‘mitochondria’, ‘protein status’ and ‘pharmacokinetics’ (Online Resource). Articles were considered eligible if they described pathophysiological mechanisms or markers underlying the causal relationship between low skeletal muscle mass and poor clinical outcomes in older cancer patients (mediating mechanisms/markers). The literature search resulted in 5078 records. After removing duplicates, 3734 articles were screened for eligibility of which 200 full-text articles were considered for this review. This literature search served as the basis for this narrative review. Specific articles were added to this narrative review based on literature from experts within the field.
